# Investigating Contributions of Canonical Transient Receptor Potential Channel 3 to Hippocampal Hyperexcitability and Seizure-Induced Neuronal Cell Death

**DOI:** 10.3390/ijms25116260

**Published:** 2024-06-06

**Authors:** Kevin D. Phelan, U Thaung Shwe, Hong Wu, Fang Zheng

**Affiliations:** 1 Department of Neurobiology and Developmental Sciences, University of Arkansas for Medical Sciences, Little Rock, AR 72205, USA; phelankevind@uams.edu; 2 Department of Pharmacology and Toxicology, University of Arkansas for Medical Sciences, Little Rock, AR 72205, USA

**Keywords:** resting membrane potential, cell resistance, excitability, paired-pulse facilitation, FluoroJade staining, stereology, TRPC3 global knockout

## Abstract

Canonical transient receptor potential channel 3 (TRPC3) is the most abundant TRPC channel in the brain and is highly expressed in all subfields of the hippocampus. Previous studies have suggested that TRPC3 channels may be involved in the hyperexcitability of hippocampal pyramidal neurons and seizures. Genetic ablation of TRPC3 channel expression reduced the intensity of pilocarpine-induced status epilepticus (SE). However, the underlying cellular mechanisms remain unexplored and the contribution of TRPC3 channels to SE-induced neurodegeneration is not determined. In this study, we investigated the contribution of TRPC3 channels to the electrophysiological properties of hippocampal pyramidal neurons and hippocampal synaptic plasticity, and the contribution of TRPC3 channels to seizure-induced neuronal cell death. We found that genetic ablation of TRPC3 expression did not alter basic electrophysiological properties of hippocampal pyramidal neurons and had a complex impact on epileptiform bursting in CA3. However, TRPC3 channels contribute significantly to long-term potentiation in CA1 and SE-induced neurodegeneration. Our results provided further support for therapeutic potential of TRPC3 inhibitors and raised new questions that need to be answered by future studies.

## 1. Introduction

The transient receptor potential (TRP) channel superfamily is a large family of cation channels encoded by 28 genes in mammals with diverse gating mechanisms and functional roles [1,2]. Canonical transient receptor potential (TRPC) channels are a subfamily of TRP that is the most closely related to the founding member of the TRP superfamily, the *drosophila trp* gene [3,4,5]. There are seven members of the mammalian TRPC family, i.e., TRPC1-7 [3,6]. Based on structural and functional similarities, the TRPC family can be divided into three subgroups: TRPC2, TRPC3/6/7, and TRPC1/4/5. TRPC2 is an important channel for olfactory function in rodents but it is a pseudogene in humans [7]. TRPC1/4/5 channels are primarily heteromeric tetramers formed by TRPC1 and other members of the subgroup [8] and are mainly activated by various G-protein coupled receptors such as metabotropic glutamate receptors [3,9,10]. TRPC3/6/7 channels are mainly homo-tetramers and are activated by diacylglycerol (DAG) directly [8,9,11]. All TRPC channels are cationic channels permeable to both monovalent cations such as Na^+^ and K^+^ and divalent cations such as Ca^2+^. They play distinct roles under physiological and pathological conditions and have been the subject of intense drug development in the last decade [12,13].

TRPC3 is the most abundant TRPC in the central nervous system (CNS) [8], and is expressed in neurons, astrocytes, and cerebrovasculature [14]. The gating mechanisms of TRPC3 channels are unique in comparison to other TRPC channels. TRPC3 channels have a higher basal activity than other TRPC channels [15]. These are also a prime example of polymodal gating. In addition to DAG, which binds to one of the lipid binding domains in TRPC3 [11,16], the TRPC3 channel can also be activated by tyrosine kinase [17], oxidative stress [18], and mechanical stretch [18]. The combination of the high abundance and complex gating made TRPC3 channels an intriguing target for drug development that may provide therapy for a host of CNS and peripheral diseases. 

The highest level of expression of TRPC3 is observed in cerebellar Purkinje neurons and hippocampal CA1-CA3 pyramidal neurons [14]. The role of TRPC3 channels in the cerebellum has been well documented. TRPC3 is essential for metabotropic type 1 glutamate (mGluR1) receptor signaling in Purkinje cells [19], and dysfunction of TRPC3 channels causes ataxia [20]. Despite similar high expression levels in hippocampal pyramidal neurons, the role of TRPC3 channels in the hippocampus has not been fully investigated. Previous studies have suggested that TRPC3 channels are critical for the signaling cascade of brain-derived neurotrophic factor (BDNF) [21,22,23]. BDNF plays a critical role in modulating synaptic plasticity in the hippocampus and contributes to learning and memory [24]. It has also been postulated as a critical contributor to many neurological disorders such as epilepsy, Huntington’s disease and Alzheimer’s disease [25,26]. Using a TRPC3 global KO mouse line, we have demonstrated that genetic ablation of TRPC3 channels reduces behavioral manifestations of seizures and the root-mean-square power of SE, indicating a significant contribution of TRPC3 channels to pilocarpine-induced status epilepticus (SE) [27]. Furthermore, the reduction in SE in TRPC3KO mice is caused by a selective attenuation of pilocarpine-induced theta activity which dominates both the pre-ictal phase and SE phase [27]. However, the cellular mechanisms of these observed changes are not known, and the impacts of these changes on SE-induced neuronal cell death are also undetermined. 

In this study, we investigated the role of TRPC3 channels in electrophysiological properties and epileptiform discharges in hippocampal pyramidal neurons. We also investigated the role of TRPC3 channels in short-term and long-term synaptic plasticity in the hippocampus. Furthermore, we investigated the impact of genetic ablation of TRPC3 channels on SE-induced neurodegeneration in the hippocampus. We found no clear changes in electrophysiological properties of hippocampal pyramidal neurons and epileptiform discharges after genetic ablation of TRPC3 channels. However, we found a significant reduction in spontaneous epileptiform discharge frequency in CA3 and a reduction in Schaffer collateral long term potentiation (LTP) in TRPC3KO mice. Furthermore, we found a significant reduction in SE-induced neuronal cell death in the hippocampus. Collectively, our results suggest that TRPC3 channels are promising targets for developing new treatment for neurological disorders. 

## 2. Results

### 2.1. Comparison of Electrophysiological Properties and Epileptiform Discharges of CA1 Pyramidal Neurons in WT and TRPC3KO Mice

To begin elucidating the cellular mechanisms through which TRPC3 channels contribute to pilocarpine-induced seizures, we investigated the role of TRPC3 channels at the cellular level in the hippocampus. Previous studies have suggested that TRPC3 channels were required for BDNF signaling in both CA1 and CA3 pyramidal neurons, and mediated a sustained cationic current that contributes to increased excitability [22,23]. 

**Figure 1:**
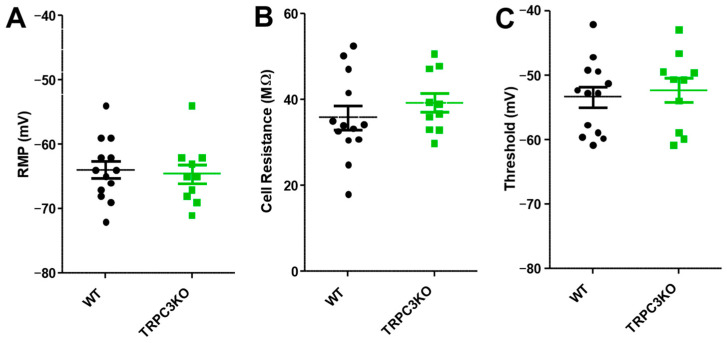
Comparison of resting membrane potential (**A**), cell input resistance (**B**), and firing threshold (**C**) of CA1 pyramidal neurons in WT (*n* = 13) and TRPC3KO mice (*n* = 10). Note that there was no significant difference between WT and TRPC3KO mice (unpaired *t*-test).

CA1 pyramidal neurons were recorded intracellularly using sharp electrodes under current clamp conditions. This approach preserved G-protein coupled receptor signaling better than patch-clamp recording methods. Each recorded pyramidal neuron was characterized by three basic electrophysiological parameters: resting membrane potential (RMP); cell resistance, which is determined by applying a small negative current pulse of 200 ms; and firing threshold, determined by manually adjusting current injection to elicit a spontaneous action potential. Based on previous publications, we expected a reduced resting membrane potential and a decrease in cell resistance and firing threshold in TRPC3KO mice. However, we found no significant differences in any of the three measured electrophysiological properties of CA1 pyramidal neurons compared to wildtype (WT) mice (Figure 1). 

**Figure 2:**
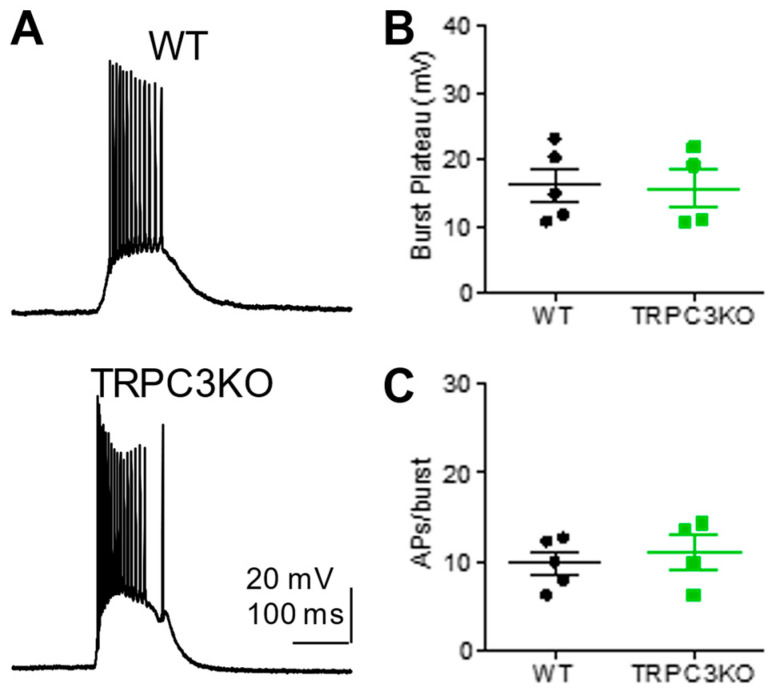
Epileptiform burst firing induced by mGluR agonist in CA1 pyramidal neurons is normal in TRPC3KO mice. (**A**) Representative current-clamp recordings showing epileptiform burst firing induced by 30 µM 1S,3R-ACPD in CA1 pyramidal neurons in adult WT and TRPC3KO mice. (**B**) The amplitude of the plateau underlying the burst is comparable in WT and TRPC3KO mice. Amplitudes were measured for three randomly selected bursts in each neuron and then averaged. Pooled data (mean ± SEM) was plotted (*n* = 5, 4 for WT and TRPC3KO mice). (**C**) The duration of each burst was quantified by the number of action potentials within each burst and three random bursts from each CA1 pyramidal neuron were analyzed to obtain the average number of spikes per burst. Pooled data (mean ± SEM) were plotted (*n* = 5, 4 for WT and TRPC3KO mice).

To determine whether TRPC3 channels in CA1 pyramidal neurons contribute to epileptiform burst firing, we applied 1S,3R-ACPD, a metabotropic glutamate receptor agonist, by bath superfusion [28]. The amplitude and duration of epileptiform bursting elicited by 1S, 3R-ACPD was comparable in WT and TRPC3KO mice (Figure 2), suggesting that TRPC3 channels are not required for epileptiform burst firing in CA1 pyramidal neurons. 

### 2.2. Comparison of Electrophysiological Properties and Epileptiform Discharges of CA3 Pyramidal Neurons in WT and TRPC3KO Mice

**Figure 3:**
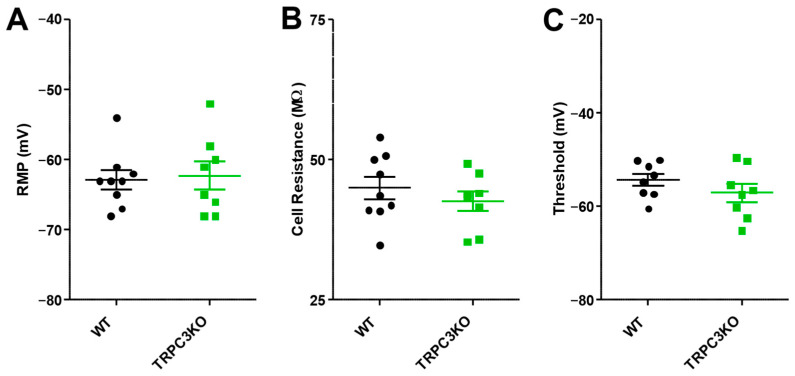
Comparison of resting membrane potential (**A**), cell input resistance (**B**), and firing threshold (**C**) of CA3 pyramidal neurons in WT (*n* = 9) and TRPC3KO mice (*n* = 8). Note that there was no significant difference between WT and TRPC3KO mice (unpaired *t*-test).

For electrophysiological experiments in the hippocampal CA3 area, we used horizontal slices to preserve entorhinal–hippocampal circuitry and CA3 pyramidal neurons were recorded intracellularly using sharp electrodes under current clamp conditions. Similar to our results from recordings of CA1 pyramidal neurons, we also found no significant differences in any of the three measured electrophysiological properties of CA3 pyramidal neurons compared to WT mice (Figure 3). 

**Figure 4:**
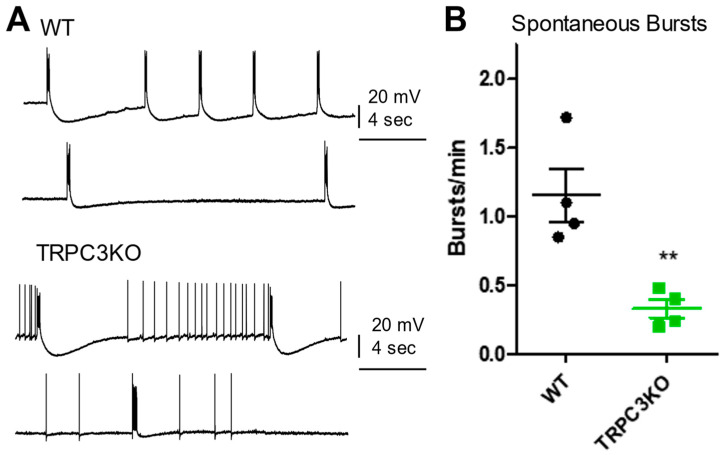
Spontaneous epileptiform burst firing induced by bicuculine in CA3 pyramidal neurons. (**A**) Representative traces of spontaneous epileptiform bursts occurred after 30 min bath application of Bicuculline in 2 WT and 2 TRPC3KO mice. (**B**) Comparison of spontaneous burst frequency after washout of bicuculine in WT (*n* = 4) and TRPC3KO mice (*n* = 4) (**: *p* < 0.01, unpaired *t*-test).

Epileptiform burst firing was elicited in CA3 pyramidal neurons by bath application of bicuculline, a competitive antagonist of GABA-A receptors [29]. These spontaneous epileptiform discharges are mediated by an enhancement in CA3 recurrent collateral synapses. As shown in Figure 4A, sustained spontaneous epileptiform bursts followed by large afterhyperpolarization potentials were observed in both WT and TRPC3KO mice following a 30 min bath superfusion of bicuculline. However, the frequency of the spontaneous bursts was significantly reduced from 1.16 burst/min in WT to 0.33 burst/min in TRPC3KO mice (*p* < 0.05, unpaired *t*-test).

**Figure 5:**
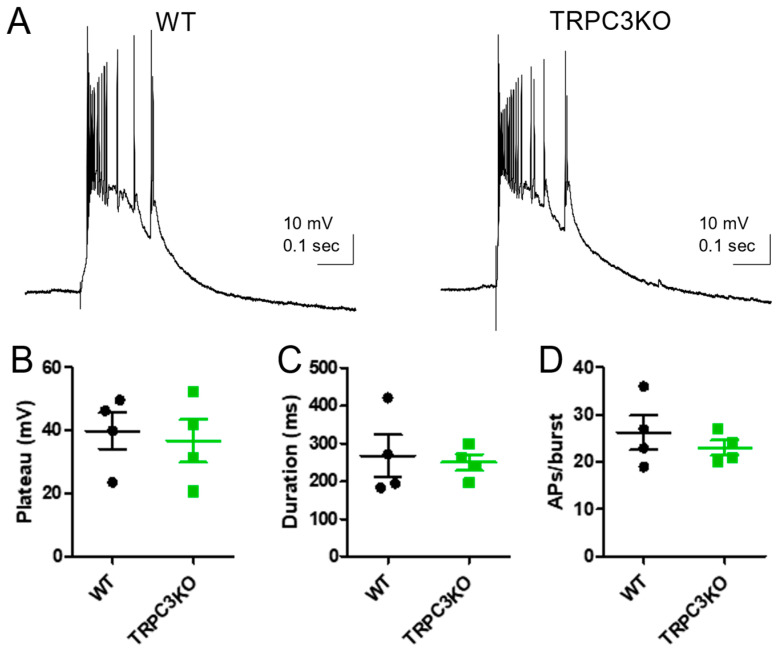
Comparison of evoked epileptiform burst discharges in WT and TRPC3KO mice. (**A**) Representative traces showing evoked epileptiform bursts by mossy fiber (MF) stimulation in CA3 pyramidal neurons after bath application of bicuculline for 30 min. (**B**–**D**) Quantitative analysis of evoked epileptiform burst firing by MF stimulations in CA3 pyramidal neurons after bath application of bicuculline for 30 min (*n* = 5, 4 for WT and TRPC3KO). There was no statistically significant difference between wildtype and TRPC3KO mice (unpaired *t*-test).

To quantify epileptiform discharges induced by bicuculine in CA3 pyramidal cells, we stimulated mossy fiber pathways to evoke those discharges. The amplitude, the duration, and the number of spikes of evoked epileptiform bursts were comparable in WT and TRPC3KO mice (Figure 5A–D). 

Collectively, we found that the frequency of spontaneous epileptiform discharges observed in CA3 pyramidal neurons after bicuculine treatment was significantly reduced. However, the amplitude, the duration and the number of spikes of evoked epileptiform bursts were not altered by genetic ablation of TRPC3 channel expression.

### 2.3. Comparison of Short-Term and Long-Term Synaptic Plasticity in WT and TRPC3KO Mice

**Figure 6:**
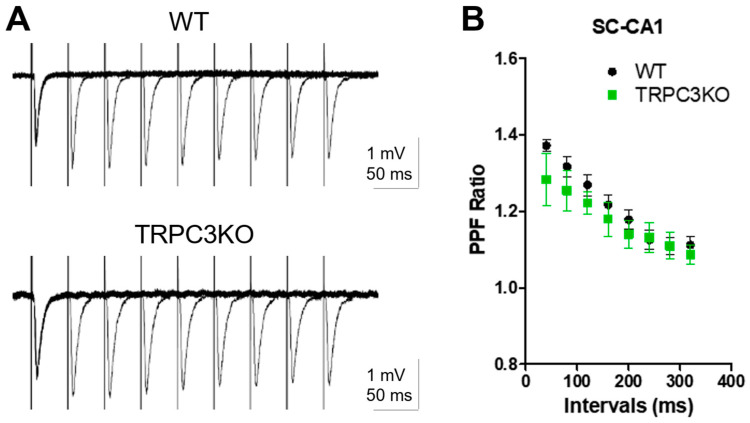
Normal paired-pulse facilitation at Schaffer collateral-CA1 synapses in TRPC3KO. (**A**) Representative traces of paired-pulse facilitation (PPF) of Schaffer collateral field EPSP in WT and TRPC3KO mice. A pair of electric stimuli with increasing intervals (40, 80, 120, 160, 200, 240, 280 and 320 ms) was delivered at 10 s intervals and the resulting pair of field EPSPs was recorded. (**B**) The averaged PPF ratios (the peak of the second EPSP over the peak of the first EPSP in each pair) and standard errors were plotted (*n* = 10, 6 for WT and TRPC3KO). Note that the peak of PPF occurs around a 40 ms interval and the subsequent decays at greater intervals. There was no statistically significant difference between wildtype and TRPC3KO mice (Two-way ANOVA).

To assess the role of TRPC3 channels in short-term synaptic plasticity, we compared paired-pulse facilitation (PPF) of Schaffer collateral-CA1 (SC-CA1) synapses in WT and TRPC3KO mice. We found that there is no significant difference between WT and TRPC3KO mice (Figure 6A,B).

**Figure 7:**
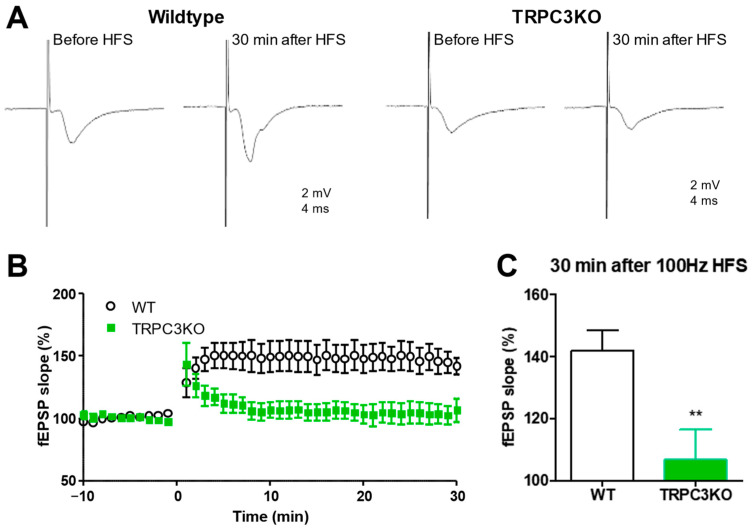
Reduced high-frequency stimuli-induced long-term potentiation at Schaffer collateral-CA1 synapses in TRPC3KO mice. (**A**) Representative traces of Schaffer collateral field EPSP recorded before and 30 min after high-frequency stimuli (HFS; 100 Hz, 1 s; repeated three times at 20-s intervals) in WT and TRPC3KO mice. Traces shown are the average of 12 consecutive recordings collected at 0.2 Hz. (**B**) Field EPSP slopes for each minute were determined by averaging 12 consecutive field EPSP recordings in each mouse, and the normalized means and standard errors were plotted (*p* < 0.01 for genotype effects, Two-way ANOVA; *n* = 14, 7 for WT and TRPC3KO mice). (**C**) Average field EPSP slope 30 min after 100 Hz HFS in WT (*n* = 14) and TRPC3KO mice (*n* = 7). Note the significantly reduced LTP in TRPC3KO mice (**: *p* < 0.01, unpaired *t*-test).

BDNF signaling plays a significant role in synaptic plasticity at the Schaffer collateral (SC)-CA1 synapse [30], and TRPC3 channels are required for BDNF signaling in the CA1 region [23]. This motivated us to determine whether TRPC3 channels are involved in synaptic plasticity at SC-CA1 synapses. We compared the high-frequency stimuli (HFS)-induced long-term potentiation (LTP) at SC-CA1 synapses in WT and TRPC3KO mice and found that the HFS-induced LTP at SC-CA1 synapses was significantly ameliorated in TRPC3KO mice (Figure 7A–C). Our results demonstrate that TRPC3 channels contribute significantly to synaptic plasticity at the SC-CA1 synapses.

### 2.4. Comparison of SE-Induced Neuronal Cell Death in WT and TRPC3KO Mice

**Figure 8:**
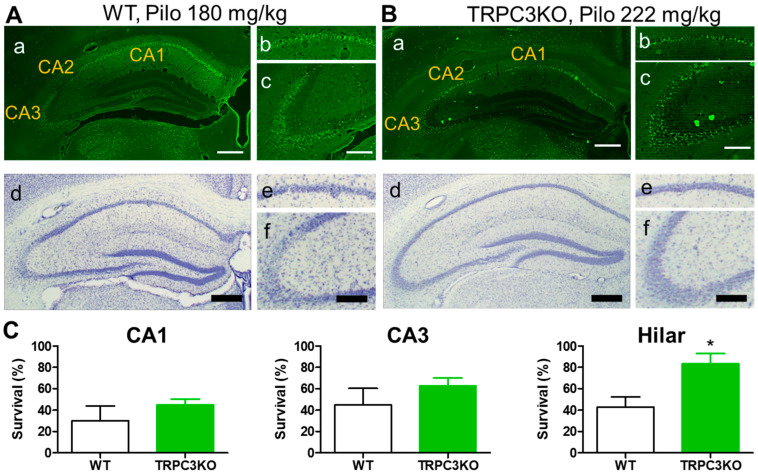
Comparison of SE-induced neuronal cell death in WT and TRPC3KO mice. (**A**,**B**) Representative images of FJC staining (**a**–**c**) and Nissl staining (**d**–**f**) of coronal hippocampal sections from WT (**A**) and TRPC3KO mice (**B**) (two-day survival; WT: 175 mg/kg pilocarpine, i.p.; TRPC3KO: 222 mg/kg pilocarpine, i.p.). CA1 (**b**,**e**) and CA3 (**c**,**f**) regions were shown at higher magnification. Scale bar: 0.2 mm for (**a**,**d**), 0.1 mm for (**b**,**c**,**e**,**f**). (**C**) Comparison of neuronal survival in the hippocampal subfields in WT (*n* = 6) and TRPC3KO mice (*n* = 9) using stereology. Note there are increases in neuronal survival in both CA1 and CA3 areas in TRPC3KO mice, but these increases are not statistically significant (*p* > 0.05, unpaired *t*-test). The increase in neuronal survival in the hilar region in TRPC3KO mice is statistically significant (*: *p* < 0.05, unpaired *t*-test).

We previously reported a significant reduction in SE intensity by genetic ablation of TRPC3 channel expression [27]. A reasonable expectation derived from this finding would be a significant reduction in SE-induced neuronal cell death in TRPC3KO mice. This motivated us to compare SE-induced neuronal cell death in WT and TRPC3KO mice. As described previously, SE-induced neuronal cell death was assessed using two methods: FluroJade staining, which showed active neuronal degeneration; and stereological analysis of Nissl-stained brain sections that quantify neuronal survival. In our previous studies, these two methods yielded consistent and complimentary results regarding SE-induced neuronal cell death. However, we found some very intriguing discrepancies in the assessment of SE-induced neuronal cell death using these two methods in TRPC3KO mice (Figure 8). As shown in Figure 8Aa,Ab, there was a clear reduction in FJC-positive neurons after SE in TRPC3KO mice. However, we were unable to detect a significant reduction in SE-induced neuronal cell death in either the CA1 or the CA3 region in TRPC3KO mice using stereology (Figure 8B). On the other hand, the density of FJC-positive neurons after SE in the hilar region appeared comparable between WT and TRPC3KO mice (Figure 8Aa,Ab), whereas stereology revealed a significant increase in neuronal survival in the hilar region. Collectively, our results suggested that TRPC3 channels contributed to SE-induced neuronal cell death in the hippocampus.

## 3. Discussion

The results presented in this study revealed an intriguing and complex picture regarding the contribution of TRPC3 channels to neuronal hyperexcitability and SE-induced neurodegeneration in the hippocampus. Although our data collectively support the notion that TRPC3 channels are a promising target for drug development, there are clear knowledge gaps in our understanding of the roles of TRPC3 channels in hyperexcitability and neurodegeneration.

The first surprising finding of this study is the lack of contributions of TRPC3 channels to epileptiform discharges of CA1 and CA3 pyramidal neurons. Given the high basal channel opening of TRPC3 [15], and previous published reports using TRPC3 selective drugs or antibodies [22,23], there was an expectation that the resting membrane potential, and the firing threshold in hippocampal pyramidal neurons should be altered in TRPC3KO mice, and these changes would be the underlying cellular mechanisms for reduced seizure intensity and reduced theta waves in TRPC3KO mice. Our results suggest that the basal activity of native TRPC3 channels in hippocampal pyramidal neurons may be very limited and TRPC3 channels have a limited direct contribution to neuronal excitability in the adult hippocampus. It should be noted that previous studies [21,23,31] were carried out using younger mice, which may account for the differences between previous reports and our results using adult mice. A slow after-depolarization potential (sADP) was observed in four of nine CA1 pyramidal neurons in the presence of 1S3R-ACPD in WT mice, whereas an sADP was only observed in one of six CA1 pyramidal neurons in the presence of 1S,3R-ACPD in TRPC3KO mice. This suggests that the TRPC3 channels may contribute to the so-called calcium-activated nonselective (CAN) current.

Our in vitro experiments in hippocampal slices also provided new insights regarding possible cellular mechanisms underlying the changes in pilocarpine-induced theta activity observed in vivo. Several previous studies have suggested a possible direct involvement of TRPC3 channels in epileptiform bursting [23,31]. Surprisingly, our data ruled out a direct involvement of TRPC3 channels in epileptiform burst firing in both CA1 and CA3 pyramidal neurons. On the other hand, the frequency of spontaneous epileptiform bursting after bath application of bicuculline in CA3 pyramidal neurons is reduced in TRPC3KO mice. A similar reduction in the frequency of spontaneous epileptiform bursting was reported in TRPC7KO mice [29]. However, genetic ablation of TRPC7 expression ameliorated evoked epileptiform bursts in CA3 pyramidal neurons [29], whereas genetic ablation of TRPC3 expression had no detectable effects on evoked epileptiform bursts. These observations suggested that the functional roles of TRPC3 and TRPC7 were distinct. We previously reported that TRPC7 channels contribute to bicuculine-induced spontaneous epileptiform bursting by mediating LTP of CA3 recurrent collateral synapses [29]. We suggest that TRPC3 channels contribute to spontaneous epileptiform burst frequency by increasing excitatory input to the CA3 region. This hypothesis is consistent with previous reports that TRPC3 channels, as part of the BDNF signaling cascade, modulate CA3 excitability [32]. We also found that the LTP at Schaffer collateral synapses is reduced in TRPC3KO mice, which is similar to the effects of genetic ablation of BDNF or trkB [30,33]. We postulate that the reduction in synaptic plasticity at Schaffer collateral synapses is a possible mechanism for the attenuation of pilocarpine-induced SE intensity in TRPC3KO mice. To test these hypotheses, cell-type-specific TRPC3KO mouse lines targeting different hippocampal neurons need to be generated and tested.

The comparison of SE-induced neuronal cell death in WT and TRPC3KO also revealed intriguing new information. TRPC3KO mice are the first mouse line in which the assessment of neuronal cell death by FJC staining and stereology yielded incongruent results. In the hippocampal CA1 subfield, FJC-positive neurons were visually reduced in TRPC3KO mice, whereas stereology indicated that the increase in CA 1 neuronal survival in TRPC3KO mice was not significant. Closer inspection of FJC staining revealed a possible explanation for this apparent contradiction. The FJC-positive neurons in WT mice were scattered evenly across the whole CA1 pyramidal cell body layer, whereas the FJC-positive neurons in TRPC3KO mice were restricted to the lower limit of the CA1 pyramidal cell body layer. This difference suggests an activation of an alternative circuitry which may increase neuronal cell death in a subpopulation of pyramidal neurons located near the lower limit of the pyramidal cell body layer. The increase in this subpopulation of pyramidal neurons offset the reduction in neuronal cell death of other pyramidal neurons. The finding that a significant increase in neuronal survival in the hilar region was not accompanied by a decrease in FJC-positive neurons is another unsolved mystery. Since the hilar region is one of the most active zones for neurogenesis, one possible explanation for our observation is that more neurons are generated in the hilar region in TRPC3 KO mice. 

In conclusion, our study provides further support for the therapeutic potential of drugs targeting TRPC3 channels and reveals new potential concerns. In support of therapeutic potentials of TRPC3 channel inhibitors, genetic ablation of TRPC3 channel expression reduces spontaneous burst firing frequency in CA3 and reduces SE-induced neuronal cell death of some subpopulations of hippocampal neurons. Overall, the most important questions that need to be answered are why and how the global knockout of TRPC3 channels alters the SE circuitry and increases neuronal cell death of certain subpopulations of hippocampal neurons. These questions have a direct impact on the therapeutic potentials of drugs targeting TRPC3 channels. Future studies are clearly needed to provide answers to this question. 

## 4. Materials and Methods

### 4.1. Electrophysiological Recordings

Transverse or horizontal slices of adult mouse brain containing the hippocampus were obtained from 2–5-month-old WT and TRPC3KO mice in a mixed 129Sv/C57Bl6 genetic background. The mice were anesthetized with ketamine (80 mg/kg) followed by decapitation. Serial 400 µm thick sections were cut with a Vibraslice (WPI, Sarasota, FL, USA), as described previously [28], and allowed to recover in oxygenated artificial cerebrospinal fluid (ACSF) for at least one hour at room temperature prior to recording. For intracellular recordings, glass microelectrodes filled with 3M sodium acetate were used as described previously [28]. Data were sampled at 5K Hz and without additional filtering. For field potential recordings, glass pipettes were pulled from filamented borosilicate glass filled with ACSF [28]. Field EPSPs were recorded in the current-clamp mode with an Axoclamp 2B amplifier (Molecular Devices, Sunnyvale, CA, USA) and digitized at 10K Hz using a model 1322A Digidata interface and pClamp 10 (Molecular Devices, Sunnyvale, CA, USA). For LTP experiments, Schaffer collaterals were stimulated with a concentric bipolar electrode with a 0.25 ms pulse, and the stimulus intensity was adjusted to produce a fEPSP approximately half the maximal amplitude. Data were analyzed using pClamp 10. 

### 4.2. Fluoro-Jade C (FJC) Staining

Two days after pilocarpine-induced seizures, mice were anesthetized with ketamine (60 mg/kg, I.M.) and then intracardially perfused with 4% paraformaldehyde as described previously [34]. The brains were removed, postfixed for at least 48 h, and then cut into 50 μm serial coronal sections using a Vibratome (Pelco 1500, Ted Pella Inc., Redding, CA, USA). Free-floating sections were stained with FJC as reported previously {28}. Four forebrain sections at least 300 µm apart that contain typical vulnerable regions (i.e., the cingulate cortex, septum, striatum, hippocampus, amygdala, piriform cortex, and thalamus) were processed to determine whether there were FJC-positive neurons. If any FJC-positive neurons were found, a complete series of 150 µm spaced sections were further stained with FJC. Images of FJC-positive sections were captured using a Coolsnap fx camera (Photometrics, Tucson, AZ, USA) mounted on an Olympus fluorescent microscope using a GFP filter.

### 4.3. Nissl Counterstaining and Stereological Analysis of Cell Death

Coronal sections (50 µm thick) were stained with 0.1% cresyl violet solution. Unbiased cell counting of the hippocampal region was obtained using Stereologer (Stereology Resource Center, Tampa, FL, USA, https://srcbiosciences.com/stereologer-software (accessed on 14 April 2024)) in serial Nissl stained sections spaced 150 µm apart extending from stereotaxic coordinates of bregma −1.3 to −2.3 mm, as described previously [34]. Surviving neurons (with stained cytoplasm and round nuclei) were counted using Stereologer with a 100× oil-immersion objective. The percentage of neuronal survival was calculated by the ratio of cell counts in pilo-treated mice over the mean cell counts of 3 untreated mice.

## Data Availability

Data supporting the reported results can be accessed by sending a request to the corresponding author directly.
